# A case report of drug–drug interaction between voriconazole and simnotrelvir/ritonavir

**DOI:** 10.3389/frabi.2025.1705987

**Published:** 2025-11-05

**Authors:** Tingting Chen, Qingquan Zhang

**Affiliations:** Department of Pharmacy, Quanzhou First Hospital, Quanzhou, Fujian, China

**Keywords:** voriconazole, simnotrelvir/ritonavir, drug–drug interaction, therapeutic drug monitoring, COVID-19

## Abstract

Co-administration of simnotrelvir/ritonavir with voriconazole should be avoided, as stated in the product insert of simnotrelvir/ritonavir, due to the anticipated decrease in the plasma concentration of voriconazole. Currently, there are no published reports regarding a pharmacokinetic interaction between simnotrelvir/ritonavir and voriconazole. We present the case of an 88-year-old man with pulmonary aspergillosis and coronavirus disease 2019 (COVID-19) co-infection treated concurrently with voriconazole and simnotrelvir/ritonavir. Prior to initiating simnotrelvir/ritonavir, two trough concentrations of voriconazole were measured, yielding values of 2.8 and 2.6 mg/L. After 2 days of co-administration with simnotrelvir/ritonavir, the voriconazole trough concentration rose to 6.0 mg/L. The voriconazole dose was subsequently reduced by 25%, and simnotrelvir/ritonavir was discontinued after completion of the standard 5-day course. A week after voriconazole dose reduction (4 days after simnotrelvir/ritonavir withdrawal), the trough concentration was measured again and was found to be 3.5 mg/L. This case indicates that the trough concentration of voriconazole increased significantly during co-administration with simnotrelvir/ritonavir. Moreover, the interaction persisted even after discontinuation of simnotrelvir/ritonavir, necessitating dynamic dose adjustments guided by therapeutic drug monitoring.

## Introduction

Simnotrelvir/ritonavir is a 3C-like (3CL) protease-targeting oral anti-severe acute respiratory syndrome coronavirus 2 (SARS-CoV-2) drug developed in China, authorized for the treatment of adult patients with mild-to-moderate coronavirus disease 2019 (COVID-19). Ritonavir, a component of simnotrelvir/ritonavir, is a strong cytochrome P450 3A (CYP3A) inhibitor and may enhance the exposure of co-administered medications. Invasive aspergillosis is common in patients with severe COVID-19 (Gioia et al., 2024), and voriconazole is one of the first-line treatment options for infection. Voriconazole is primarily metabolized in the liver and acts both as an inhibitor and a substrate of CYP2C9, CYP2C19, and CYP3A4. It exhibits a high potential for drug–drug interactions with other substrates, inhibitors, or inducers of CYP enzymes (Theuretzbacher et al., 2024).Voriconazole exhibits nonlinear pharmacokinetics and substantial inter-individual variability, necessitating therapeutic drug monitoring (TDM), as recommended by guidelines (Ashbee et al., 2014; Chen et al., 2018; Takesue et al., 2022), to optimize its efficacy and safety. The target trough concentration of voriconazole was defined as 1–5.5 mg/L for the present case according to the guidelines.

Currently, there are no published reports regarding a pharmacokinetic interaction between simnotrelvir/ritonavir and voriconazole. We present the case of an 88-year-old man with pulmonary aspergillosis and COVID-19 co-infection treated concurrently with voriconazole and simnotrelvir/ritonavir. The trough concentration of voriconazole increased significantly during co-administration with simnotrelvir/ritonavir, and the interaction persisted even after discontinuation of simnotrelvir/ritonavir.

## Case

An 88-year-old male patient weighing 50 kg was admitted to our hospital on May 19, 2025, with a chief complaint of cough, expectoration, and wheezing for over 2 months. A month prior to this hospital admission, the patient was diagnosed with invasive pulmonary aspergillosis and was started on antifungal therapy with voriconazole 200 mg q12h orally (loading dose, 400 mg q12h). The patient adhered to the voriconazole regimen regularly after discharge. Physical examination on this admission showed: temperature, 36.6°C; heart rate, 84 beats/min; respiratory rate, 26 breaths/min; and blood pressure, 118/75 mmHg. Arterial blood gas analysis showed: pH, 7.441; PaCO_2_, 45.8 mmHg; PaO_2_, 67.7 mmHg; and SpO_2_, 96%. On auscultation, bilateral coarse breath sounds were heard, with diminished breath sounds in the lower lung fields. No significant dry or moist rales were detected, and the remainder of the physical examination was unremarkable. Laboratory tests showed: white blood cell count, 9.40 × 10^9^/L; neutrophils, 6.31 × 10^9^/L; hemoglobin, 121 g/L; C-reactive protein, 2.25 mg/L; procalcitonin, <0.04 ng/ml; serum creatinine, 50.7 μmol/L; blood urea nitrogen, 8.08 mmol/L; albumin, 34.0 g/L; total bilirubin, 12.2 μmol/L; aspartate aminotransferase, 22 U/L; and alanine aminotransferase, 18 U/L. Chest CT revealed inflammatory changes in both lungs. The admission diagnoses were pulmonary aspergillosis and hypertension.

**Figure 1:**
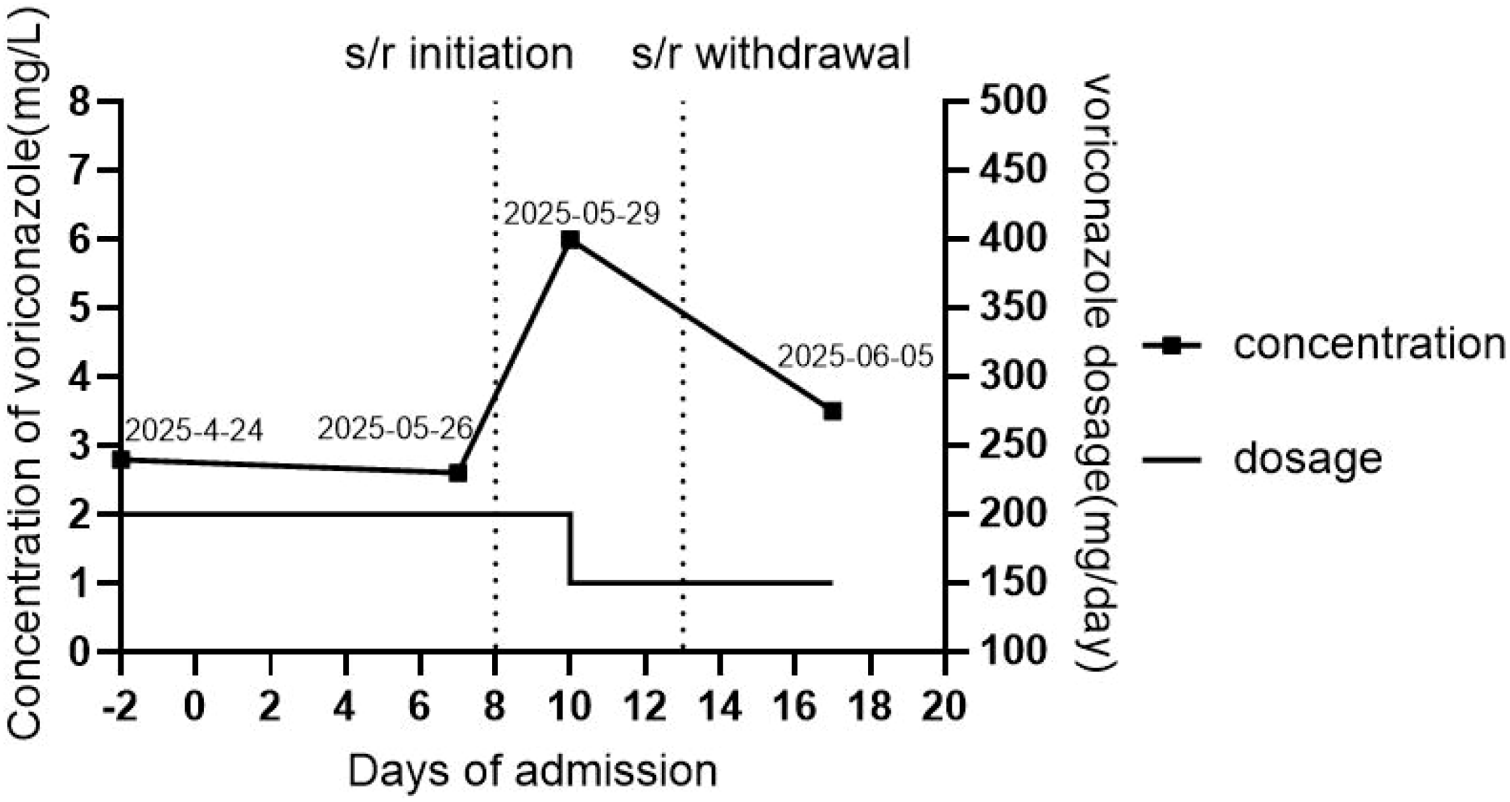
Changes in the trough concentration of voriconazole in the patient. Day “−2” denotes the day before the hospital admission, while day “0” denotes the day of the current admission. “*s/r*” indicates simnotrelvir/ritonavir, which was administered from day 8 to day 13.

During the current admission, oral voriconazole (200 mg q12h) was continued for antifungal treatment. The initial voriconazole trough concentration, measured using the enzyme-multiplied immunoassay technique (EMIT) 30 min prior to the fifth dose (April 19, 2025), was 2.8 mg/L. A repeated voriconazole trough concentration on May 26 was 2.6 mg/L. On May 27, the patient experienced increased wheezing. Arterial blood gas analysis showed: pH, 7.415; PaCO_2_, 51.3 mmHg; PaO_2_, 65.9 mmHg; and SpO_2_, 93.8%. A SARS-CoV-2 nucleic acid test was positive, and oral simnotrelvir/ritonavir (750 mg/100 mg every 12 h) was started for antiviral treatment. Later that day, the patient’s wheezing worsened and SpO_2_ decreased to approximately 85%. Accordingly, meropenem 1 g q8h was added empirically. On May 29, the voriconazole trough concentration increased to 6.0 mg/L, leading to a dose reduction to 150 mg q12h orally. No adverse drug reactions were observed at this elevated concentration. The simnotrelvir/ritonavir regimen was completed on May 31 after a 5-day course. By June 5, a week after voriconazole dose reduction (4 days after simnotrelvir/ritonavir withdrawal), the voriconazole trough concentration decreased to 3.5 mg/L. On June 10, the patient maintained SpO_2_ above 97% with noninvasive ventilation, showed no dyspnea at rest, and remained afebrile. Meropenem was discontinued after a 14-day course of therapy, with the absence of identified pathogens. The patient was discharged accordingly. **Figure 1** shows the serial changes in the trough concentrations of voriconazole in this patient.

## Discussion

Simnotrelvir/ritonavir was conditionally approved in January 2023 in China for the treatment of adult patients with mild-to-moderate COVID-19 (Cao et al., 2024).Ritonavir is a potent inhibitor of CYP3A4, while simnotrelvir is a substrate of CYP3A. By inhibiting CYP3A4, ritonavir significantly slows the metabolic clearance of simnotrelvir *in vivo*, thereby increasing and prolonging its plasma concentration to exert therapeutic effects. Voriconazole is a broad-spectrum antifungal agent and is one of the first-line treatment options for invasive aspergillosis (Koehler et al., 2021). Co-administration of simnotrelvir/ritonavir with voriconazole should be avoided, as stated in the product insert of simnotrelvir/ritonavir, due to the anticipated decrease in the plasma concentration of voriconazole (Liu et al., 2007). Thus far, there have been no clinical data describing the drug–drug interactions between simnotrelvir/ritonavir and voriconazole.

The study by Ye et al. (2025) suggested that simnotrelvir/ritonavir may be co-administered with CYP3A4 inhibitors. Liu et al. (2007), in their study in healthy volunteers, reported a significant reduction in voriconazole exposure when co-administered with ritonavir. In contrast, other studies have observed an increasing trend in the voriconazole trough concentrations during co-administration with nirmatrelvir/ritonavir, a finding inconsistent with the prescribing information of nirmatrelvir/ritonavir (Shi et al., 2025; López-Hernández et al., 2025). In addition, another study indicated that the inhibitory effects of ritonavir may persist even after its discontinuation (Katzenmaier et al., 2011).

In this case, the patient received oral voriconazole 200 mg q12h regularly prior to initiating combination therapy with simnotrelvir/ritonavir. Two trough concentrations of voriconazole were measured: 2.8 and 2.6 mg/L. After 2 days of co-administration with simnotrelvir/ritonavir, the trough concentration of voriconazole increased significantly to 6.0 mg/L. In accordance with guideline recommendations, the voriconazole dose was empirically reduced by 25% to 150 mg q12h orally, with repeat TDM recommended due to its nonlinear pharmacokinetics. Simnotrelvir/ritonavir was discontinued after 3 days of co-administration. After 7 days of the voriconazole dose reduction (4 days after discontinuation of simnotrelvir/ritonavir), the voriconazole trough concentration was 3.5 mg/L, a value still higher than the pre-combination baseline. During this hospitalization, the trough concentrations of voriconazole were monitored at three time points: before, during, and after co-administration with simnotrelvir/ritonavir. The patient’s liver and kidney function were normal throughout this period, and no other medications with known interactions with voriconazole were administered. The TDM results demonstrated the effect of simnotrelvir/ritonavir on voriconazole exposure.

In addition to drug–drug interactions, CYP2C19 gene polymorphism influences the initial steady-state trough concentration of voriconazole (Lamoureux et al., 2016). A systematic review demonstrated that CYP2C19 poor metabolizers have a significantly higher voriconazole trough concentration than extensive metabolizers (Li et al., 2016). Similarly, a prospective multicenter study in Spain reported that rapid and ultra-rapid metabolizers exhibit lower trough concentrations compared with intermediate or normal metabolizers (Blanco-Dorado et al., 2020). The study by Wang et al. (2024) showed that the magnitude of interactions between voriconazole and nirmatrelvir/ritonavir was influenced by the CYP2C19 phenotype. The study by Liu et al. (2007) demonstrated that the effect of ritonavir on the voriconazole trough concentrations was dose-dependent. However, at both 400 and 100 mg bid ritonavir doses, a number of subjects exhibited elevated voriconazole trough concentrations. The authors suggested that this increase was likely due to CYP2C19 deficiency. Another study showed that short-term co-administration of ritonavir increased voriconazole exposure across all CYP2C19 genotypes, with this effect being particularly pronounced in CYP2C19 poor metabolizers (Mikus et al., 2006).

In the present case, CYP2C19 genotyping was not performed for this patient. However, the patient’s voriconazole trough concentration was within the target range with standard-dose therapy, suggesting that the CYP2C19 genotype did not significantly influence the initial steady-state trough concentration. Nevertheless, the genotype may have influenced the magnitude of interactions between voriconazole and simnotrelvir/ritonavir.

This case indicates that the trough concentration of voriconazole increased significantly during co-administration with simnotrelvir/ritonavir. Moreover, the interaction persisted even after discontinuation of simnotrelvir/ritonavir, necessitating dynamic dose adjustments guided by TDM. However, this case report has several limitations. Firstly, the CYP2C19 genotype, a known factor affecting voriconazole concentrations, was not determined. Secondly, the generalizability of the findings is inherently limited by the single-case design.

## Data Availability

The original contributions presented in the study are included in the article/supplementary material. Further inquiries can be directed to the corresponding author.
